# Supplementary material for: Empirical methods for controlling false positives and estimating confidence in ChIP-Seq peaks
Source: BMC Bioinformatics. 2008 Dec 5;9:523. doi: 10.1186/1471-2105-9-523 (PMC2628906; doi:10.1186/1471-2105-9-523)
Supplement: Additional File 1 — A variety of html documents from the USeq web site detailing the available applications, their best usage, output file type descriptions, command line menus, etc. [file 1471-2105-9-523-S1.zip › USeqUserGuides/JavaDoc.zip!/JavaDoc/bioroot/Preference.html]

Preference


|  |  |  |
| --- | --- | --- |
| **Overview** \| **Package** \| **Class** \| **Use** \| **Tree** \| **Deprecated** \| **Index** \| **Help** | |  |
| **PREV CLASS**   **NEXT CLASS** | **FRAMES**    **NO FRAMES**     **All Classes** |  |
| SUMMARY: NESTED \| FIELD \| CONSTR \| METHOD | DETAIL: FIELD \| CONSTR \| METHOD |  |


---


## bioroot Class Preference

```
java.lang.Object
  bioroot.Preference
```

**Direct Known Subclasses:**: StrainPreference

---

``` public class Preference extends java.lang.Object ```

**Author:**
:   nix

---

**Constructor Summary**

`Preference()`

`Preference(java.lang.String databaseConcat)`
  
          databaseConcat = visibility : orderBy: labGroups : features separated by colons,
labGroups and feature lines separated by semi-colons


| **Method Summary** | |
| --- | --- |
| `java.lang.String[]` | `fetchNames()`             returns a String[] of names |
| `Feature[]` | `getFeatures()` |
| `java.lang.String[]` | `getLabGroups()` |
| `int` | `getNumberToDisplay()` |
| `java.lang.String` | `getOrderBy()` |
| `java.lang.String[]` | `getVisibilities(DBUtil bioRoot, UserBean userBean)` |
| `java.lang.String` | `getVisibility()` |
| `boolean` | `isOrdered()` |
| `java.lang.String` | `makeConcatination()`             Makes a visibility: orderBy: labGroups: features concat. |
| `void` | `setFeatures(Feature[] features)` |
| `void` | `setLabGroups(java.lang.String[] labGroups)` |
| `void` | `setNumberToDisplay(int n)` |
| `void` | `setOrderBy(java.lang.String orderBy)` |
| `void` | `setOrdered(boolean ordered)` |
| `void` | `setVisibility(java.lang.String visibility)` |

**Methods inherited from class java.lang.Object**

`equals, getClass, hashCode, notify, notifyAll, toString, wait, wait, wait`

**Constructor Detail**

### Preference

```
public Preference()
```

---


### Preference

```
public Preference(java.lang.String databaseConcat)
```

:   databaseConcat = visibility : orderBy: labGroups : features separated by colons,
    labGroups and feature lines separated by semi-colons


**Method Detail**

### fetchNames

```
public java.lang.String[] fetchNames()
```

:   returns a String[] of names

---


### makeConcatination

```
public java.lang.String makeConcatination()
```

:   Makes a visibility: orderBy: labGroups: features concat.

---


### getFeatures

```
public Feature[] getFeatures()
```

---


### setFeatures

```
public void setFeatures(Feature[] features)
```

---


### getLabGroups

```
public java.lang.String[] getLabGroups()
```

---


### setLabGroups

```
public void setLabGroups(java.lang.String[] labGroups)
```

---


### getOrderBy

```
public java.lang.String getOrderBy()
```

---


### setOrderBy

```
public void setOrderBy(java.lang.String orderBy)
```

---


### getVisibilities

```
public java.lang.String[] getVisibilities(DBUtil bioRoot,
                                          UserBean userBean)
```

---


### getVisibility

```
public java.lang.String getVisibility()
```

---


### setVisibility

```
public void setVisibility(java.lang.String visibility)
```

---


### isOrdered

```
public boolean isOrdered()
```

---


### setOrdered

```
public void setOrdered(boolean ordered)
```

---


### getNumberToDisplay

```
public int getNumberToDisplay()
```

---


### setNumberToDisplay

```
public void setNumberToDisplay(int n)
```


---


|  |  |  |
| --- | --- | --- |
| **Overview** \| **Package** \| **Class** \| **Use** \| **Tree** \| **Deprecated** \| **Index** \| **Help** | |  |
| **PREV CLASS**   **NEXT CLASS** | **FRAMES**    **NO FRAMES**     **All Classes** |  |
| SUMMARY: NESTED \| FIELD \| CONSTR \| METHOD | DETAIL: FIELD \| CONSTR \| METHOD |  |


---
